# Neuron-specific enolase level is a useful biomarker for distinguishing amyotrophic lateral sclerosis from cervical spondylotic myelopathy

**DOI:** 10.1038/s41598-021-02310-2

**Published:** 2021-11-24

**Authors:** Akihiro Tsukahara, Takafumi Hosokawa, Daisuke Nishioka, Takuya Kotani, Shimon Ishida, Tohru Takeuchi, Fumiharu Kimura, Shigeki Arawaka

**Affiliations:** 1 Division of Neurology, Department of Internal Medicine IV, Osaka Medical and Pharmaceutical University, 2-7 Daigaku-machi, Takatsuki, Osaka 569-8686 Japan; 2 Department of Medical Statistics, Research and Development Center, Osaka Medical and Pharmaceutical University, Takatsuki, Osaka Japan; 3 Division of Rheumatology, Department of Internal Medicine IV, Osaka Medical and Pharmaceutical University, Takatsuki, Osaka Japan; 4 Department of Internal Medicine, Osaka Medical and Pharmaceutical University Mishima-Minami Hospital, Takatsuki, Osaka Japan

**Keywords:** Neuroscience, Biomarkers

## Abstract

The current study aimed to evaluate whether cerebrospinal fluid (CSF) neuron-specific enolase (NSE) levels are elevated in amyotrophic lateral sclerosis (ALS) and are effective in distinguishing ALS from cervical spondylotic myelopathy (CSM). We retrospectively evaluated 45 patients with ALS, 23 with CSM, 28 controls, and 10 with Parkinson’s disease (PD) who underwent analysis of CSF NSE levels. The control group comprised patients aged above 45 years who underwent lumbar puncture because of suspected neurological disorders that were ruled out after extensive investigations. CSF NSE levels were evaluated using the electro-chemiluminescent immunoassay. The ALS group had significantly higher CSF NSE levels than the CSM and control groups (*P* < 0.001 for both comparisons). The CSM, control, and PD groups did not significantly differ in terms of CSF NSE levels. A receiver-operating characteristic curve analysis was performed to assess the diagnostic value of CSF NSE levels in distinguishing ALS from CSM. The area under the curve for CSF NSE levels was 0.86. The optimal cutoff value was 17.7 ng/mL, with a specificity of 87% and a sensitivity of 80%. Hence, CSF NSE levels are elevated in ALS and are effective in distinguishing ALS from CSM.

## Introduction

Amyotrophic lateral sclerosis (ALS) is a progressive and fatal disease characterized by the neurodegeneration of both upper and lower motor neurons. The pathogenesis of the condition is unclear, and its diagnosis is made clinically^1^. As there are no specific tests for ALS, a detailed set of diagnostic criteria has been established^2^. However, some patients with ALS do not fulfill the clinical criteria on ALS particularly at the early stage, and they are misdiagnosed with different neurological and medical disorders^1,3^. Importantly, a misdiagnosis of cervical spondylotic myelopathy (CSM) is an important problem. ALS is most commonly misdiagnosed as CSM^4,5^. Patients with ALS present with focal muscle weakness and atrophy without bulbar symptoms at the early stage of the disease^3,6^, which is similar to cervical spondylosis (CS). Among CS, ALS characterized by lower limb spasm but without radicular pain might be easy to distinguish from cervical spondylotic radiculopathy, but not from CSM. Further, a misdiagnosis of CSM may lead to unnecessary surgery^7,8^ and subsequently more rapid deterioration because some patients with ALS experience accelerated disease progression after operation^9,10^. A novel tool is required to differentiate ALS from CSM.

Neuron-specific enolase (NSE) is a glycolytic enzyme predominantly observed in neurons and endocrine cells^11^. The intraneuronal NSE is secreted into the extracellular space after substantial neuronal damage. However, NSE is not physically secreted. Therefore, an elevated CSF NSE level mainly reflects neuronal damage^12^. In fact, this phenomenon is observed in different conditions associated with central nervous system damage, such as traumatic brain injury^13^, traumatic spinal cord injury^14^, acute brain infarction^15,16^, Parkinson’s disease (PD)^17^, Alzheimer’s disease^18^ multiple system atrophy^19^, bacterial meningoencephalitis^20^, and Creutzfeldt–Jakob disease^21^. However, thus far, there have been no reports, at least those written in English, about CSF NSE levels in ALS.

Therefore, this study investigated whether CSF NSE levels are elevated in ALS and whether they are a useful biomarker for distinguishing ALS from CSM.

## Results

### Characteristics of patients

The characteristics of the four groups at time of CSF sampling are shown in Table 1. One-third of patients with ALS did not present with bulbar symptoms, and about one-half had cervical cord compression on magnetic resonance imaging (MRI). All patients finally fulfilled the criteria on definite, probably, or PLS ALS. However, approximately one-half did not meet the criteria at time of CSF sampling. There were no significant differences in terms of age and the proportion of male patients between the ALS, CSM, control, and PD groups. There was no significant difference in terms of disease duration between the ALS, CSM, and PD groups. All patients with CSM had cervical cord compression on MRI according to the inclusion criteria of this study. Further, there were significant differences in the proportion of patients with cervical cord compression between the four groups (*P* < 0.001).

**Table 1:** Characteristics of the ALS, CSM, control, and PD groups at the time of CSF sampling.

	ALS group (n = 45)	CSM group (n = 23)	Control group (n = 28)	PD group (n = 10)	*P* value
Age (years), mean ± SD	70.2 ± 8.5	67.4 ± 10.0	67.0 ± 14.5	67.5 ± 14.1	NS
Male sex, n (%)	21 (47)	18 (78)	12 (43)	5 (50)	NS
Disease duration (months), mean ± SD	14.8 ± 11.1	14.8 ± 14.4	NA	20.3 ± 20.0	NS
Bulbar symptoms, n (%)	30 (67)	NA	NA	NA	
ALSFRS-R, mean ± SD	36.1 ± 8.2	NA	NA	NA	
Cervical cord compression on MRI, n (%)	21/41 (51)	23/23 (100)	7/21 (33)	2/5 (40)	< 0.001
El Escorial category					
Definite, n (%)	7 (16)	NA	NA	NA	
Probable, n (%)	9 (20)	NA	NA	NA	
PLS, n (%)	7 (16)	NA	NA	NA	
Possible, n (%)	13 (29)	NA	NA	NA	
Suspected, n (%)	9 (20)	NA	NA	NA	

*ALS* amyotrophic lateral sclerosis, *ALSFRS-R* revised ALS functional rating scale, *CSF* cerebrospinal fluid, *CSM* cervical spondylotic myelopathy, *NA* not applicable, *NS* not significant, *PD* Parkinson’s disease, *PLS* probable laboratory-supported.

### CSF NSE levels

**Figure 1:**
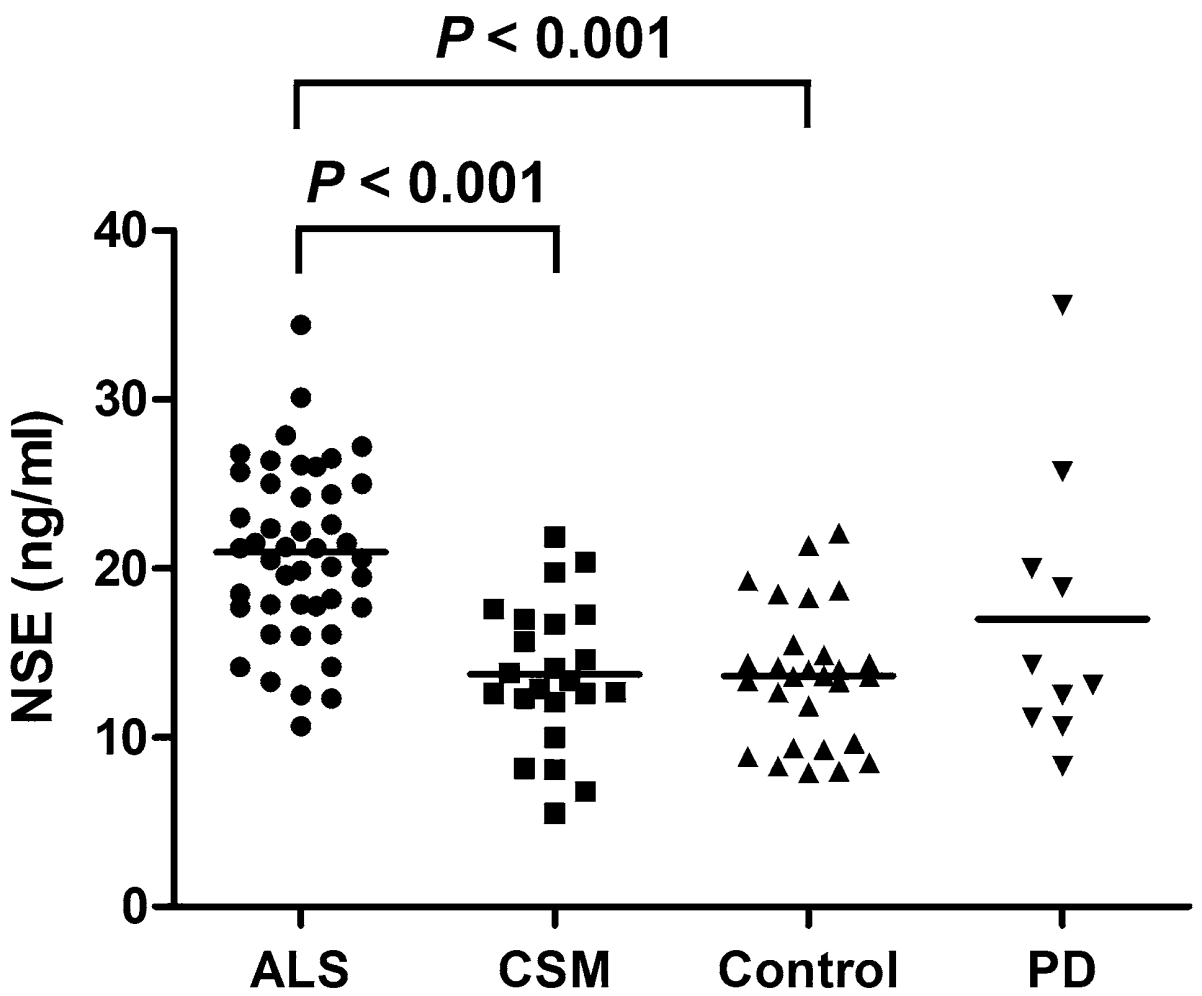
CSF NSE levels of the ALS, CSM, control, and PD groups. The solid line represents the mean CSF NSE levels of each group. *ALS* amyotrophic lateral sclerosis, *CSF* cerebrospinal fluid, *CSM* cervical spondylotic myelopathy, *NSE* neuron-specific enolase, *PD* Parkinson’s disease.

**Figure 2:**
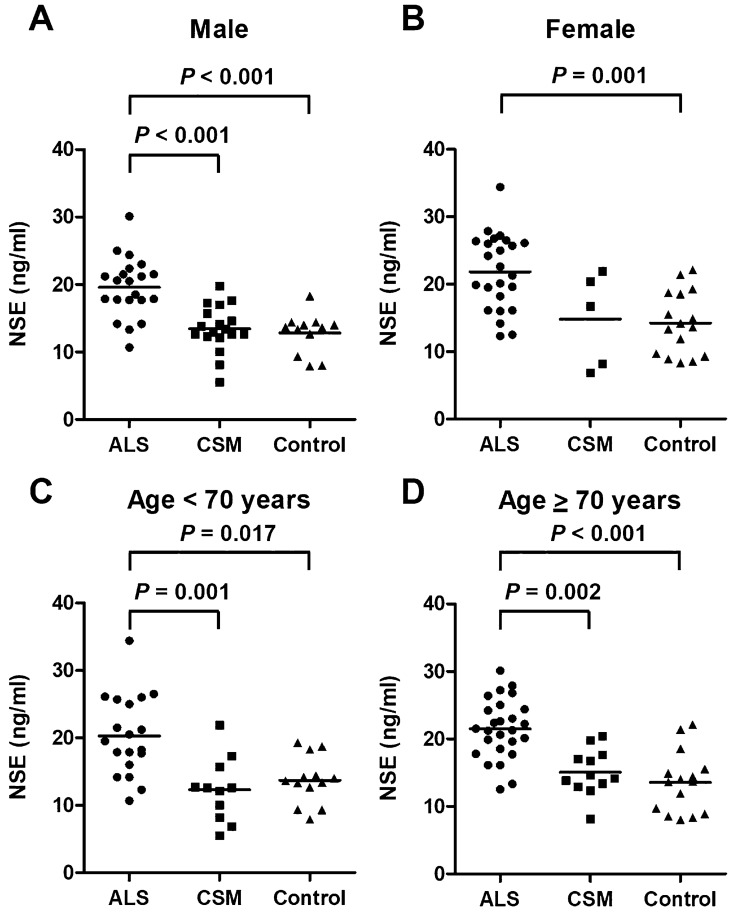
CSF NSE levels of the ALS, CSM, and control groups based on the subgroup analyses of (**A**) male and (**B**) female patients, (**C**) those aged < 70 years, and (**D**) those aged ≥ 70 years. The solid line represents the mean CSF NSE levels of each group. *ALS* amyotrophic lateral sclerosis, *CSF* cerebrospinal fluid, *CSM* cervical spondylotic myelopathy, *NSE* neuron-specific enolase.

**Figure 3:**
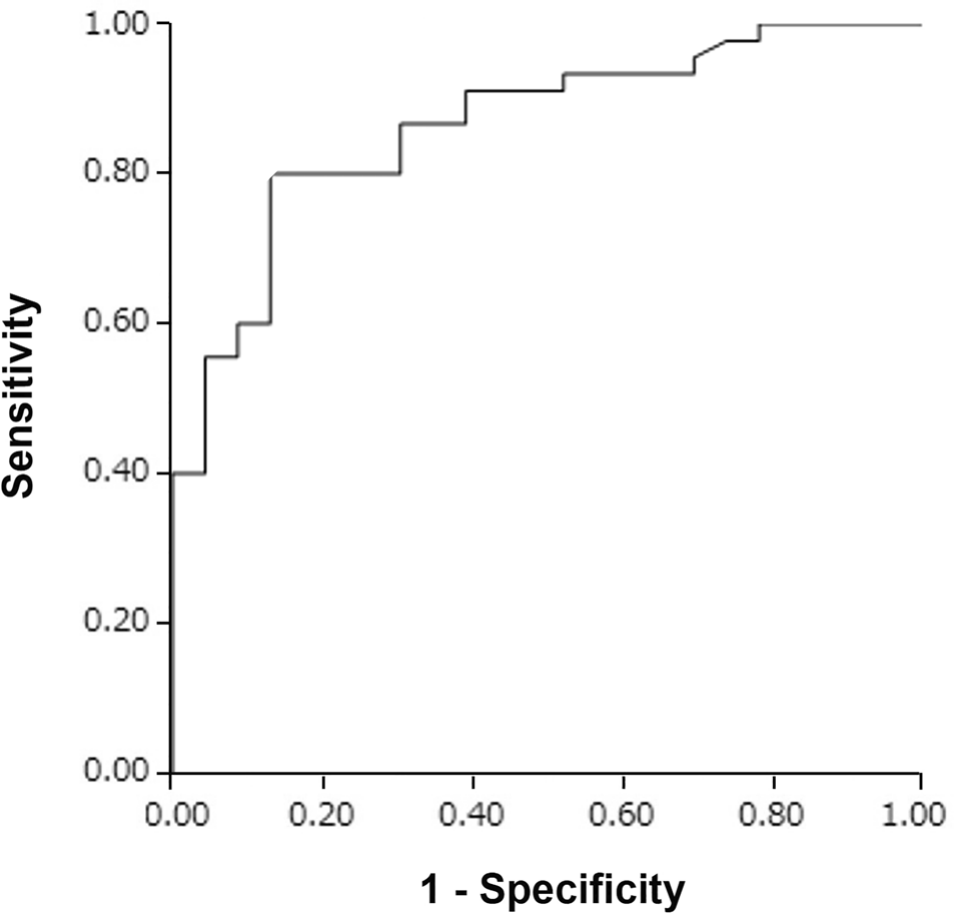
Receiver operating characteristic curves for distinguishing ALS from CSM based on CSF NSE levels. *ALS* amyotrophic lateral sclerosis, *CSF* cerebrospinal fluid, *CSM* cervical spondylotic myelopathy, *NSE* neuron-specific enolase.

The ALS group (mean ± standard deviation: 21.0 ± 5.1 ng/mL) had significantly higher CSF NSE levels than the CSM (13.7 ± 4.3 ng/mL, *P* < 0.001) and control (13.6 ± 4.0 ng/mL, *P* < 0.001) groups (Fig. 1). There was no significant difference in terms of CSF NSE levels between the CSM, control, and PD groups. To control the confounding effects of age and sex, we further performed several subgroup analyses of male and female patients and those aged < 70 and ≥ 70 years, respectively (Fig. 2), although the PD group was excluded from the further analysis because of its small sample size. In the subgroup analyses of men and those aged < 70 and ≥ 70 years, the ALS group had significantly higher CSF NSE levels than the CSM group (*P* < 0.001 for male patients, *P* = 0.001 for those aged < 70 years, and *P* = 0.002 for those aged ≥ 70 years) and the control group (*P* < 0.001 for male patients, *P* = 0.017 for those aged < 70 years, and *P* < 0.001 for those aged ≥ 70 years). The CSF NSE levels did not differ between the CSM and control groups. In a subgroup analysis of women, the ALS group had significantly higher CSF NSE levels than the control group (*P* = 0.001). Moreover, the ALS group had higher CSF NSE levels than the CSM group (*P* = 0.133) although the results did not significantly differ possibly due to the small sample size. An ROC curve analysis was performed to assess the diagnostic value of CSF NSE levels in distinguishing ALS from CSM (Fig. 3). The AUC of CSF NSE levels was 0.86. The optimal cutoff value was 17.7 ng/mL, with a specificity of 87% and sensitivity of 80%.

**Figure 4:**
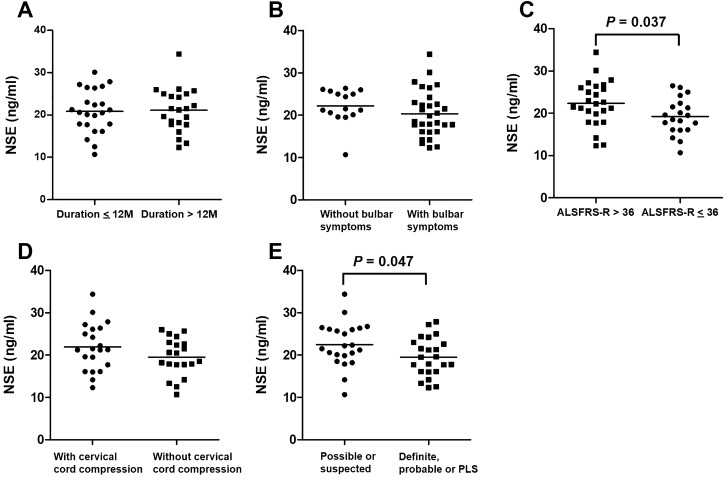
Associations between CSF NSE levels and clinical characteristics in patients with ALS at the time of CSF sampling. The CSF NSE levels of patients (**A**) with a disease duration of ≤ 12 and > 12 months, (**B**) those with and without bulbar symptoms, (**C**) those with an ALSFRS-R score of > 36 and ≤ 36, (**D**) those with and without cervical cord compression on MRI, and (**E**) those with possible or suspected ALS and definite, probable, or PLS ALS. The solid line represents the mean CSF NSE levels of each group. *ALS* amyotrophic lateral sclerosis, *ALSFRS-R* revised ALS functional rating scale score, *CSF* cerebrospinal fluid, *CSM* cervical spondylotic myelopathy, *NSE* neuron-specific enolase, *PLS* probable laboratory-supported.

Figure 4 shows the associations between CSF NSE levels and clinical characteristics in patients with ALS at the time of CSF sampling. Patients with possible or suspected ALS (22.5 ± 5.2 ng/mL) had significantly higher CSF NSE levels than those with definite, probable, or PLS ALS (19.5 ± 4.5 ng/mL, *P* = 0.046). Moreover, the CSF NSE levels were significantly higher in patients with ALS who had an ALSFRS-R score of > 36 (22.4 ± 5.3 ng/mL) than in those with an ALSFRS-R score of ≤ 36 (19.2 ± 4.3 ng/mL, *P* = 0.037). There were no significant differences in terms of CSF NSE levels between patients with and without bulbar symptoms; those who had a disease duration of ≤ 12 and > 12 months; and those with and without cervical cord compression on MRI. ROC curve analysis was performed to assess the diagnostic value of CSF NSE levels in distinguishing ALS with several features from CSM. The AUCs of CSF NSE levels were 0.91 in ALS without bulbar symptoms, 0.87 in ALS with cervical cord compression, and 0.91 in ALS that do not fulfill the criteria on definite, probable, or PLS.

## Discussion

The primary finding of this study is that CSF NSE levels are elevated in ALS. Based on a previous study, they are influenced by age and sex^22^. Moreover, this research showed elevated CSF NSE levels in ALS via the subgroup analyses of male and female patients and those aged < 70 and ≥ 70 years, which were performed to control the confounding effects of age and sex. To the best of our knowledge, this study, at least among those written in English, first showed elevated CSF NSE levels in ALS. Another main finding is that CSF NSE levels are higher in ALS than in CSM; therefore, they are useful in distinguishing ALS from CSM. In relation to the finding, the CSF NSE levels were not elevated in CSM. In the literature, the CSF NSE levels in CSM are controversial. That is, a previous report showed high CSF NSE levels in CSM^23^. Meanwhile, another revealed normal levels^24^. Taken together, CSF NSE levels in CSM may not be as elevated as those in ALS. Hence, they can be used to distinguish ALS from CSM.

There are several explanations why the ALS group had higher CSF NSE levels than not only the control but also CSM groups, even though NSE is generally a non-specific marker of neural damage^12^. First, the difference in CSF NSE levels may reflect different degrees of neural damage. A widespread and aggressive neural damage in ALS can result in significantly elevated CSF NSE levels. However, a limited and non-aggressive neural damage in CSM may not. Second, differences in CSF NSE levels can reflect varying affected areas. CSM patients have degenerations in the anterior horn cells of the spinal cord. On the other hand, ALS patients have degenerations not only in the anterior horn cells of the spinal cord but also the Betz cells of the brain cortex and the motor nuclei of the brainstem. Notably, the brain cortex, which could be involved in ALS but not in CSM, has been reported to have high NSE levels^25^. Third, differences in CSF NSE levels may reflect varying pathologic processes. Although NSE is expressed in neuronal cells and is secreted into the extracellular space after substantial damage of neuronal cells as mentioned previously, NSE has been reported to be included in not only neuronal cells but also microglia^26^, astrocytes^27^, and oligodendrocytes^28^. Moreover, NSE expression and activity are markedly increased in neuronal and glial cells under several pathologic processes. That is, NSE may not be a non-specific marker of neural damage, but may play a role in several pathologic processes in which not only neuronal cells but only glial cells may be involved, and the mechanism might occur in ALS. In fact, NSE has been increased and involved in pathologic processes such as neuroinflammation, particularly in the expression of pro-inflammatory cytokines and the proliferation of inflammatory glial cells^25,29^. In addition, the importance of neuroinflammation in ALS has been reported^30^. However, specific pathologic processes related to elevated CSF NSE levels in ALS is not addressed. Moreover, the pathologic process related to the elevation of CSF NSE levels might not be triggered by toxic factors in the CSF of patients with ALS. Askanas et al. treated cultured rat motor neurons with CSF from patients with ALS and measured the NSE levels in the neurons primarily as an index of neuronal health^31^. The authors found that treatment with CSF from patients with ALS did not alter NSE levels in cultured rat motor neurons; thus, they concluded that the study failed to demonstrate the presence of toxic factors in the CSF from patients with ALS that would influence rat motor neurons. We further speculate that they also failed to demonstrate the presence of toxic factors in the CSF from patients with ALS which would trigger pathologic changes related to increased NSE expression in rat motor neurons.

Spinal MRI is a useful but insufficient tool for distinguishing ALS from CSM. The reason is that ALS patients must frequently exhibit degenerative disk disease and spondylosis of the cervical spine in MRI imaging regardless of symptomatic or asymptomatic, because both ALS and CSM preferentially affect individuals of middle of old age. In fact, approximately half of the patients with ALS were reported to have concomitant CS^32^. Moreover, asymptomatic disk disease and spondylosis of the cervical spine in MRI imaging were reported to be frequent in individuals of middle of old age^33^. The findings are also consistent with our results that one-half of patients with ALS had cervical cord compression on MRI regardless of symptomatic or asymptomatic. Subsequently, the misdiagnosis of CSM has been reported to be frequent among patients with ALS^4,5^, and concomitant ALS might be missed in those with CSM. In terms of treatment approaches, a misdiagnosis of CSM in patients with ALS is crucial because it may lead to unnecessary surgery and subsequent more rapid deterioration as mentioned in introduction. Notably, overlooking concomitant ALS in patients with CSM might be also crucial. Surgical treatment should be carefully considered in patients with CSM and concomitant ALS, even in those with symptomatic CSM and concomitant ALS. Although surgery for CS results in temporary alleviation, the major cause of motor symptoms is usually attributed to ALS in those patients and the motor symptoms might deteriorate more rapidly in some cases^32^. Therefore, CSF NSE level is a useful marker to determine the presence of ALS without the potential influence of CSM.

Distinguishing ALS from CSM is challenging, particularly when patients with ALS present with cervical cord compression on MRI and they do not experience bulbar symptoms and do not fulfill the criteria on ALS. In this study, patients with ALS and such features had significantly higher CSF NSE levels than those with ALS without such features, or the CSF NSE levels of the former group was as high as those of the latter group. In detail, patients with ALS who do not fulfil the criteria on definite, probable, or PLS ALS had significantly higher CSF NSE levels than those who fulfilled the criteria. The CSF NSE levels of patients with ALS with cervical cord compression was as high as those of patients with ALS without compression. Moreover, the CSF NSE levels of patients with ALS without bulbar symptoms was as high as those of patients with ALS with the symptoms. Consequently, the diagnostic values of CSF NSE levels in distinguishing ALS with such features from CSM were higher or as high as those of CSF NSE levels in distinguishing whole ALS from CSM. In cases in which patients with ALS are challenging to distinguish from those with CSM, CSF NSE can be used. Hence, it may be an effective biomarker.

The reason why patients with ALS who do not fulfil the criteria had significantly higher CSF NSE levels than those who fulfilled the criteria is uncertain. However, it could be explained by a hypothesis that CSF NSE levels might decrease with disease progression at a certain stage because it could be accompanied by a decreased number of motor neurons, which might be the source of CSF NSE. Notably, the hypothesis could also explain our findings that patients with mild ALS had higher CSF NSE levels than other patients.

CSF NSE levels have been reported to be elevated in PD^17^. In this study, while mean CSF NSE levels were higher in PD group than in CSM and control groups and lower in PD groups than in ALS group, these differences did not reach statistical significance. Because these findings may be influenced by small sample size of the patients especially with PD, further studies are needed.

Our study had several limitations. First, it had a small sample size and was retrospective in nature. Hence, further large prospective studies should be conducted. Second, the control group only comprised unhealthy patients who underwent lumbar puncture, which is an invasive test, because of suspected neurological disorders that were ruled out after extensive investigations.

CSF NSE levels are elevated in ALS. Further, they can effectively distinguish ALS from CSM and prevent the misdiagnosis of CSM in patients with ALS. Thus, unnecessary surgery and subsequent rapid deterioration may be prevented. Notably, numerous physicians including those in general medical institutions can benefit from the use of this biomarker in daily clinical practice because NSE is a common tumor marker for diseases including small lung cancer and can be measured in general medical institutions. In addition, because elevated CSF NSE levels in ALS may reflect a specific pathologic process, this finding could provide new perspectives regarding the understanding of ALS pathogenesis and could facilitate the development of appropriate treatments.

## Materials and methods

### Patients

We retrospectively evaluated 45 patients with ALS, 23 with CSM, 10 with PD, and 28 controls who were admitted to Osaka Medical and Pharmaceutical University Hospital and who underwent lumbar puncture and subsequent analysis of CSF NSE levels from January 2014 to January 2021. Patients were diagnosed with definite, probable, or probable laboratory-supported (PLS) ALS according to the revised El Escorial criteria^2^. Since the criteria include EMG findings of fibrillation potentials, positive sharp waves, large motor unit potentials, reduced interference pattern, and unstable motor unit potentials, we performed EMG in all ALS patients and carefully check the presence or absence of these findings. The diagnosis of ALS based on the criteria requires the absence of electrophysiological or pathological evidence of other disease processes that might explain the signs of lower and/or upper motor neuron degeneration. Therefore, we performed nerve conduction study in all ALS patients. The diagnosis also requires the absence of neuroimaging evidence of other disease processes that might explain the observed clinical and electrophysiological signs, we performed brain MRI in all but one ALS patient in whom we performed brain CT at least, and cervical MRI in 21 of the 41 ALS patients. Those with ALS and concomitant CSM were classified under the ALS group. CSM was diagnosed based on the presence of myelopathic symptoms, such as limb numbness, problems with fine motor skills, and gait disturbance, and radiologic cervical cord compression in the stenotic canal, which is correlated with the patients’ symptoms. The control group comprised patients aged above 45 years who underwent lumbar puncture due to suspected neurological disorders that were ruled out after extensive investigations. As NSE was reported to be a biomarker of PD^17^, we included a group of 10 PD patients. Not only controls and patients diagnosed with ALS, CSM, and PD at the time of CSF sampling but also those diagnosed at a later time (up to February 2021) were included. Patients with other concomitant neurological or neuromuscular disorders were excluded.

**Figure 5:**
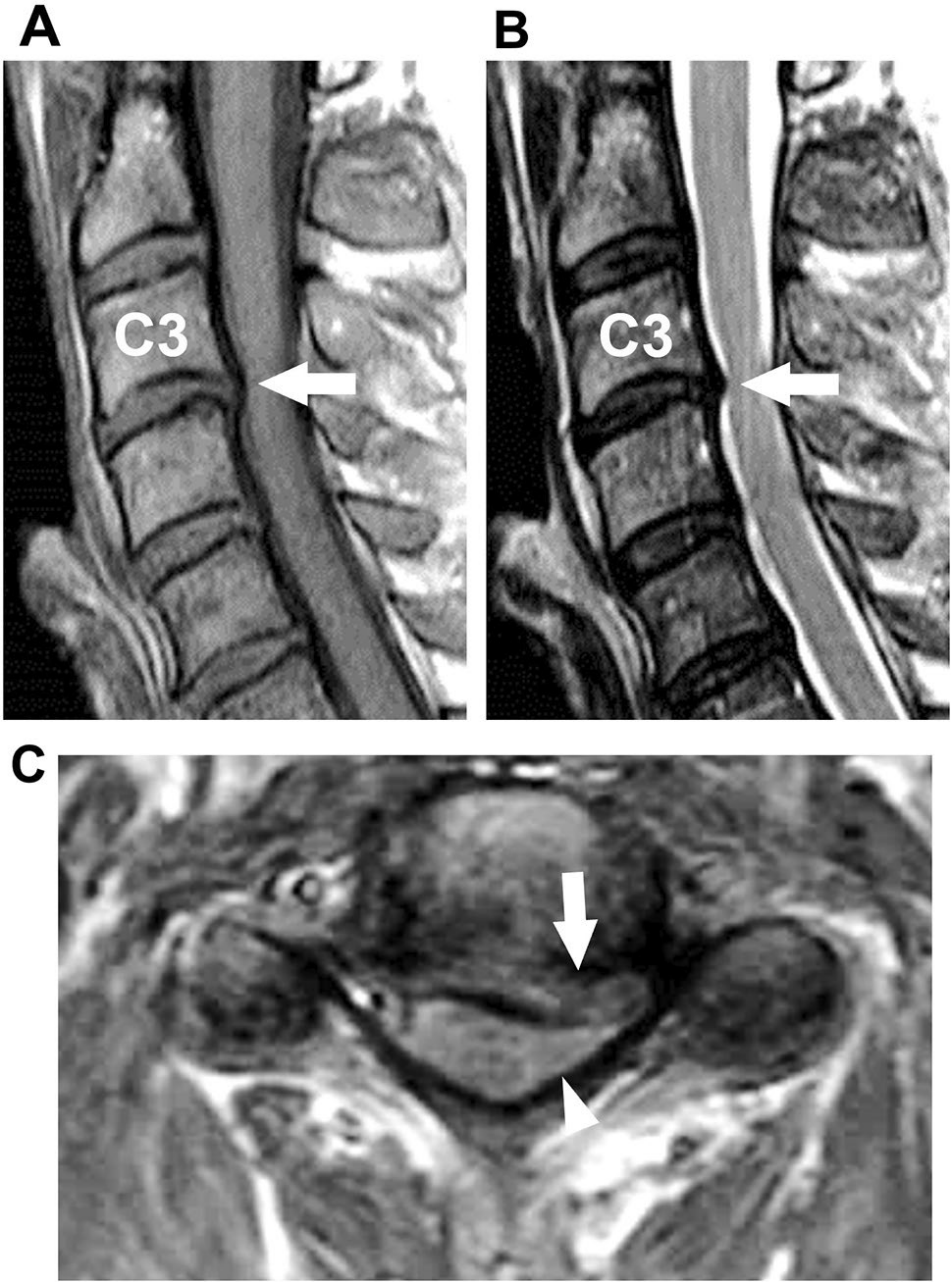
Cervical cord compression on MRI. (**A**) T1- and (**B**) T2-weighted sagittal images of 50-year-old man with CSM show posterolateral disk protrusion at C3/4 levels producing concave defect in cervical cord (arrow). (**C**) T1-weighed axial image at C3/4 level shows posterolateral disk protrusion (arrow) compressing spinal cord (arrowhead). *CSM* cervical spondylotic myelopathy.

Information about age, sex, disease duration, neurological symptoms, disability, spinal MRI findings, and ALS categories according to the El Escorial criteria were collected at the time of CSF sampling. Based on the presence of a concave defect in the cervical cord caused by the impingement of the disc or osseous material on MRI regardless of defect degree and its symptoms, cervical cord compression was considered (Fig. 5). Disability associated with ALS was determined using the Revised ALS Functional Rating Scale Score (ALSFRS-R), which has a maximum of 48 points. Lower scores represent a more severe disease stage^34^.

This study was conducted according to the 2013 Helsinki Declaration, and the Osaka Medical and Pharmaceutical University Ethics Committee approved the study protocol and the need for informed consent was waived because this was a retrospective study and the data were collected without individual patient identifiers (Approval number # 2020-189).

### CSF NSE analysis

CSF samples were collected via lumbar puncture. Then, they were immediately brought to the laboratory for analysis. CSF NSE levels were evaluated using the electro-chemiluminescent immunoassay performed by SRL (Tokyo, Japan). The detection limit was 0.1 ng/mL.

### Statistical analysis

The Mann–Whitney U test was used to assess differences in continuous variables between two groups. The Kruskal–Wallis test, followed by the Dunn’s multiple comparison test, was utilized to evaluate differences between three or four groups. Meanwhile, the chi-square test was applied to examine categorical variables. To investigate the accuracy of biomarkers in differentiating ALS from CSM, a receiver operating characteristic (ROC) curve analysis was performed by calculating the area under the ROC curve (AUC). The optimal cutoff value was chosen using the maximized Youden index. The values were expressed as mean ± standard deviation, and a *P* value of < 0.05 was considered statistically significant. All analyses were performed using the JMP software version 15.0 (SAS Institute Inc., Cary, NC, the USA).

## Data Availability

The datasets generated during and/or analyzed during the current study are available from the corresponding author on reasonable request.
